# The application of nanodiscs in membrane protein drug discovery & development and drug delivery

**DOI:** 10.3389/fchem.2024.1444801

**Published:** 2024-09-18

**Authors:** Yingkui Dong, Huan Tang, Han Dai, Hongxin Zhao, Junfeng Wang

**Affiliations:** ^1^ High Magnetic Field Laboratory, Key Laboratory of High Magnetic Field and Ion Beam Physical Biology, Hefei Institutes of Physical Science, Chinese Academy of Sciences, Hefei, Anhui, China; ^2^ Institute of Physical Science and Information Technology, Anhui University, Hefei, Anhui, China; ^3^ Hefei China Science Longwood Biological Technology Co., Ltd, Hefei, Anhui, China; ^4^ University of Science and Technology of China, Hefei, Anhui, China

**Keywords:** nanodisc (ND), membrane protein, drug discovery & development, drug delievery, membrane scaffold protein (MSP)

## Abstract

The phospholipid bilayer nanodiscs (LNDs), as a rapidly-developing tool in recent years, provide a natural bio-memebrane environment to maintain the native conformation and functions of membrane proteins as well as a versatile delivery vehicle for a variety of hydrophobic and hydrophilic drugs. We have seen unprecedented advantages of phospholipid bilayer nanodiscs in membrane protein structure characterization, biochemical and physiological studies of membrane proteins, membrane environment studies, drug discovery & development, and drug delivery. Many previous reviews have been mainly focused on the advantages of nanodiscs in membrane protein researches, but few have touched upon the importance and potential application of nanodiscs in pharmaceutical industries. This review will provide general description of the structural characteristics, advantages, classification, and applications of phospholipid nanodiscs, with particular focus on nanodisc-enabled membrane protein drug discovery & development as well as drug delivery.

## 1 Introduction

Membrane proteins are proteins found in cell membranes, either at the surface or on intracellular organelles. Integral membrane proteins such as receptors and ion channels span the membrane. Peripheral membrane proteins, for example, some signalling proteins, are tethered by an anchor, via a fatty acid, prenyl group, glycophosphatidylinositol (GPI) or a hydrophobic protein patch (Telser, 2002). Cells must rely on membrane proteins to exchange substances, energy, and information with the surrounding environment (von Heijne, 2007; Xie and Dalbey, 2008). The most classic classification of membrane proteins is based on their location in the lipid bilayer into two categories (Vit and Petrak, 2017; Levental and Lyman, 2023): peripheral membrane proteins (PMPs) and integral membrane proteins (IMPs), also known as transmembrane proteins (Martin and Sawyer, 2019).

PMPs use a variety of different mechanisms to associate with biological membranes in their microenvironment. They can “nest” in the membrane, interact with the membrane interior through their hydrophobic structure, or simply interact with the polar head groups of phospholipids (Monje-Galvan and Klauda, 2016). PMPs often play key roles in metabolic pathways, making them attractive targets for the treatment of diseases ranging from tuberculosis (Yano et al., 2014) and cancer (Moreno-Vivián et al., 1999; Sparacino-Watkins et al., 2014; Saxena et al., 2018; Tomczyk et al., 2018) to parasitic infections (Ebiloma et al., 2019). However, their highly amphipathic nature and dependence on lipid interactions also limit the study of their structure and function, as well as the ability to target them in computer-aided drug design (Costeira-Paulo et al., 2018).

Integral membrane proteins are important to all living cells and perform many key functions. They transport ions, metabolites, and larger molecules across the membrane (Vit and Petrak, 2017), are responsible for sending and receiving chemical signals, transmitting electrical impulses, connecting cells to each other, and anchoring other proteins to specific locations in the cell (Chitwood and Hegde, 2019). Other functions include regulating intracellular vesicle transport, controlling membrane lipid composition, and organizing and maintaining the shape of organelles and cells themselves. These roles also make integral membrane proteins attractive targets for treating various diseases such as metabolic diseases (Amaravadi et al., 2019). About half of the currently approved human medical drugs target integral membrane proteins (Chen et al., 2023). Similar to PMPs, integral membrane proteins have higher hydrophobicity, which makes it more difficult to study membrane proteins in an experimental environment compared to soluble proteins. Therefore, it is particularly necessary to develop, utilize, and develop biological membrane simulation systems that can give membrane proteins physiological conformations and functions.

**FIGURE 1:**
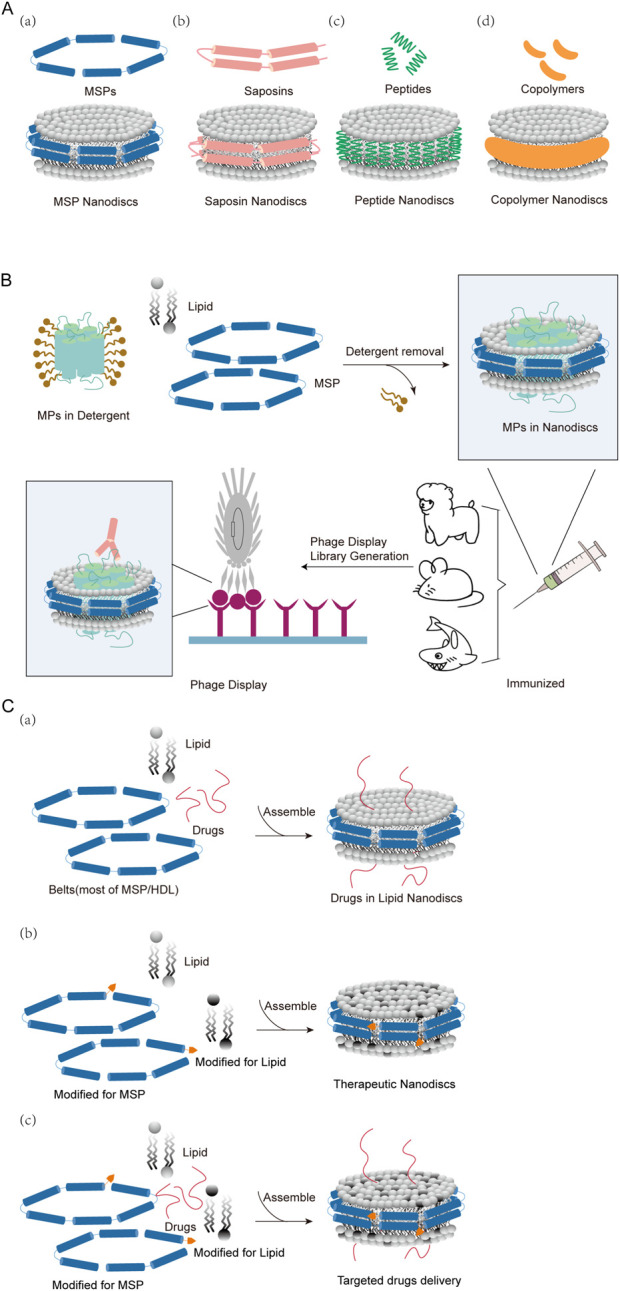
Classification of nanodiscs, membrane protein drug discovery, and drug incorporation. **(A)** Four main categories of nanodiscs: **(a)** MSP and MSP nanodiscs **(b)** Saposin and Saposin nanodiscs **(c)** Small peptides and peptide nanodiscs **(d)** Copolymers and copolymer nanodiscs. **(B)** Schematic diagram of antibody discovery using phage display technology for MSP nanodiscs **(C) (a)** Some drugs are covalently bound to nanodiscs **(b)** The lipids or belts of nanodiscs are modified to achieve coupling or targeting functions **(c)** Some targeted drugs are inserted into modified nanodiscs.

In 1998, Sligar and colleagues first demonstrated the nanodiscs (NDs) technology in a study targeting liver microsomes-cytochrome P450 oxidoreductase (CYP450) (Bayburt et al., 1998). The first lipid nanodisc was a membrane mimetic made of a phospholipid bilayer surrounded by high-density lipoprotein (HDL). Since then, the diversity of nanodiscs has expanded to include “ribbon” structures formed by membrane scaffold proteins (MSP), saposins, peptide, or copolymers to maintain the integrity of the nanodisc structure (Figure 1A) (Young, 2023). All of these nanodiscs are self-assembled, nanosized, and disc-shaped phospholipid bilayer structures, therefore also called phospholipid nanodiscs (LNDs). Lipid Nanodiscs, as a new type of nanomaterial, can simulate the double-layer structure of cell membranes and provide important tools for membrane protein researches, drug delivery, vaccine development, biosensors and other fields (Borch and Hamann, 2009). Lipid nanodiscs are formed through the cooperative assembly of phospholipid bilayer membranes and stabilizers (such as MSP proteins or synthetic polymers) and can exist stably in aqueous solutions. Lipid molecules self-assemble in the aqueous phase to form a bilayer structure, and stabilizers form a stable nanodisc structure by wrapping the edges of the lipid bilayer to prevent its disassembly. This double-layer membrane structure can effectively simulate the environment of the cell membrane, allowing biological macromolecules such as membrane proteins to maintain activity and function in a close-to-natural state. The diameter of lipid nanodiscs is generally between 10 and 20 nm and the thickness is about 4–5 nm. Their size and composition can be controlled by adjusting the ratio and type of lipids and stabilizers. Its unique structure makes it an ideal platform for studying membrane protein function and drug delivery.

In membrane protein research, Micelle stands as the most classic system, but its lack of a phospholipid membrane environment may lead to the denaturation of certain membrane proteins (Milic and Veprintsev, 2015). Liposome and Bicelle systems, though commonly employed in membrane-related research, pose challenges such as larger molecular weight, poor uniformity, and instability (Barbosa-Barros et al., 2012; Durr et al., 2013; Zou et al., 2022). One of the main advantages of nanodisc technology is that it does not contain detergent molecules and can maintain its integrity and shape after dilution (Sligar and Denisov, 2021). This overcomes the shortcomings of micelles or bicelles. Compared with micelles or bielles, LNDs provides a membrane environment that is more like that of natural cell membranes (Hagn et al., 2013; Raghuraman et al., 2019; Das and Raghuraman, 2021). Compared with liposomes, it has a relatively fixed size and a range of controllable sizes and dimensions (Boldog et al., 2006). In addition, the oligomeric states of membrane proteins can be separated by controlling the size of nanodiscs (Boldog et al., 2006).

## 2 Classification of nanodiscs

Depending on the stabilizer, lipid nanodiscs can be divided into different types: membrane scaffold protein (MSP) nanodiscs (MSPND), (Denisov et al., 2004), saposin nanodiscs (Frauenfeld et al., 2016), peptide nanodiscs (Carlson et al., 2018), and copolymer nanodiscs using synthetic polymers such as styrene maleic acid (SMA) (Orwick et al., 2012) (Figure 1A). The original MSPNDs have undergone significant technological development over the past decade, whereas saposin nanodiscs, peptide nanodiscs and copolymer nanodiscs are recently emerging nanodisc alternatives.

### 2.1 MSP nanodiscs

MSP nanodiscs use amphipathic membrane scaffold protein (MSP) as stabilizers to wrap a stable discoidal phospholipid bilayer and embedded transmembrane proteins to form a nanodisc (Figure 1Aa). MSP is generally a truncated form of apolipoprotein (apo) A-I constituent of high-density lipoproteins, which is wrapped around a small piece of phospholipid bilayer to form disc-shaped nanodiscs. MSP provides a hydrophobic surface facing the hydrophobic tail of the lipid, as well as a hydrophilic surface on the outside. This arrangement makes the nanodiscs highly soluble in aqueous solutions. In this process, in addition to the detergent added for purifying membrane proteins, a large amount of additional detergent needs to be added, and then the detergent is removed by adding bio-beads during the assembly process (Bayburt et al., 2002; Denisov et al., 2004). Thus, Once assembled into nanodiscs, membrane proteins can be kept in solution without detergents (Hagn et al., 2013; Yu et al., 2023). MSP nanodiscs range in size from 7–17 nm. The size is determined by the membrane scaffold protein used. MSP nanodiscs can be used to characterize membrane proteins expressed in prokaryotes and eukaryotes, including structures such as various transporters, ion channels, and G protein-coupled receptors (GPCRs) (Coudray et al., 2020; Zhang et al., 2021).

### 2.2 Saposin nanodiscs

The saposin protein family contains four family members saposin A–D with molecular weight around 10 kDa). saposin A has been mainly used for saposin nanodisc assembly. Frauenfeld et al. have successfully used saposin protein as scaffold proteins to reconstitute a number of membrane proteins in a phospholipid environment (Frauenfeld et al., 2016). The saposin protein, phospholipids, and membrane proteins self-assemble into saposin nanodisc. The advantage of saposin nanodiscs is their versatile adaptation to the various sizes of the membrane protein, without the requirement to screen for different membrane scaffold protein constructs and protein-to-lipid ratios. The development of saposin nanodiscs is relatively recent, and therefore their applications are mainly limited to structure-based analytical techniques such as NMR and Cryo-EM. However, saposin nanodiscs offer some unique applications for each of these techniques. In a solution-based NMR study, three different membrane proteins were incorporated into saposin nanodiscs: bacterial outer membrane protein X (OmpX), sensory receptor rhodopsin II (pSRII), and β1-adrenergic receptor (β1AR) (Chien et al., 2017). Cryo-EM studies have characterized several different receptor membrane proteins reconstituted in saposin nanodiscs: protein-coupled oligopeptide transporters (POTs) (Frauenfeld et al., 2016), bacterial efflux transporter (AcrB) (Du et al., 2020), smoothened receptor (SMO) (Zhang et al., 2022), and various nicotinic receptors (Rahman et al., 2020; Noviello et al., 2021; Rahman et al., 2021), among others.

### 2.3 Peptide nanodiscs

Another alternative to MSP nanodiscs is peptide nanodiscs, or peptidisc (Carlson et al., 2018). The reconstruction of the peptide nanodisc is based on an apolipoprotein A-I mimetic peptide - nanodisc scaffold peptide (NSP) (Islam et al., 2018). The initial version of NSP was a scaffold peptide with a sequence of 37 amino acids, and shorter versions up to 18 amino acids were subsequently developed. Compared to MSP nanodiscs or Saposin nanodiscs, the process of reconstituting membrane proteins into peptide nanodiscs appears to be more streamlined and does not require the addition of specific phospholipids. The nanodisc scaffold peptide itself is highly flexible and can adapt to the size of the transmembrane region of membrane proteins. Peptide nanodiscs can also be used to reconstitute membrane proteins immediately after extraction from lipid bilayers with mild detergents. This feature is highly beneficial for stabilizing and purifying multi-subunit protein complexes that would otherwise dissociate after prolonged exposure to detergents. Although peptidisc can be used to purify affinity-tagged membrane proteins under detergent-free conditions, there are no reports of high-resolution structural data for membrane proteins or complexes purified from peptid libraries (Young et al., 2020). Furthermore, although the “peptid library” approach for membrane protein characterization has been widely applied to bacterial membrane proteins, its application to eukaryotic membrane proteins has not yet been reported (Young et al., 2022; Young et al., 2023).

### 2.4 Copolymer nanodiscs

Copolymer nanodiscs extract the membrane proteins in their native state directly from the cell membranes and incorporate membrane protein in their endogenous phospholipids. Assembly of synthetic copolymer nanodiscs begins with intact cells. In this process, synthetic polymers are used that serve dual functions.Membrane proteins from native membranes can be spontaneously encapsulated directly from the membrane into nanosized copolymer nanodiscs. This produces a nanoslice of the native membrane that is excised and stabilized by the synthetic polymer rings (Knowles et al., 2009; Jamshad et al., 2015; Maier et al., 2023). Natural cellular phospholipids are then used to form nanodisc structures around membrane proteins. The polymer acts as both a solubilizer and a stabilizer. Therefore, no additional detergent is required. This type of nanodisc uses synthetic copolymers such as styrene-maleic acid (SMA) copolymer, diisobutylene maleic acid (DIBMA) copolymer, or polymethacrylate (PMA) copolymer as stabilizers to stabilize the lipid bilayer membrane (Zhu et al., 2022). SMA or other polymers interact with lipid molecules and self-assemble into stable nanodiscs in aqueous solution. The synthetic polymer used to synthesize copolymer nanodiscs is non-protein and has higher purity than MSP nanodiscs. Synthetic copolymer nanodiscs are mainly used in membrane protein research, drug delivery, and biosensors. The size of synthetic copolymer nanodiscs is variable, and the main factor that determines the diameter of synthetic copolymer nanodiscs is the size of the membrane protein complex. SMA nanodiscs have been successfully used to purify and characterize integral membrane proteins from bacteria and eukaryotic systems. Membrane proteins reconstituted with SMA nanodiscs are suitable for high-resolution structural characterization by cryo-EM, as well as receptor-ligand binding assays and activity assays (Postis et al., 2015; Parmar et al., 2018; Swainsbury et al., 2023).

Table 1 shows the applications of some different types of nanodiscs. It can be seen that MSP nanodiscs and Peptide nanodiscs are the most widely used. There are also some studies using SMA nanodiscs. For Saposin nanodiscs, most of the research is still focused on the structural study of membrane proteins (Elzoghby et al., 2023).

**TABLE 1:** Some examples of NDs used in drugs development and drugs delivery applications.

Nanodiscs type	Inserted	Applications	References
MSP nanodiscs	Hydrophobic drugs&Cabazitaxel	Cancer chemotherapy	Pandey et al. (2021)
MSP nanodiscs (Lipids modified)	—	Antibody-conjugated NDs for targeted cancer imaging	Wong et al. (2020)
MSP nanodiscs	Influenza virus M2 protein	Antibody Discovery	Yu et al. (2023)
apoA I-stabilized Nanodiscs (like MSP nanodiscs)	Amphotericin B	Anti-fungal drug delivery	Cho et al. (2017)
ApoJ-stabilized Nanodiscs (like MSP nanodiscs)	—	Alzheimer’s disease treatment	Fernandez-de-Retana et al. (2017)
Peptide nanodiscs	Adpgk & CM9 peptides	Cancer vaccine	Najafabadi et al. (2021)
Peptide nanodiscs	DTX/CpG	ICD inducer	Kadiyala et al. (2019)
Peptide nanodiscs (KT peptide modified)	T0901317	LXR agonist for diabetic nephropathy	He et al. (2022)
Peptide nanodiscs	ML355	Antiplatelet drug	He et al. (2020)
SMA nanodiscs	Bacterial Outer Membrane	Antibacterial Vaccination	Noh et al. (2022)

CBZ, cabazitaxel; DTX, docetaxel; KT, kidney targeting; ICD, immunogenic cell death.

## 3 Lipid nanodiscs for membrane protein drug discovery & development

Membrane proteins are a class of amphipathic, highly insoluble proteins in aqueous solutions, but they are often important targets for antibody discovery, such as GPCRs, ion channels, etc. *In vitro* studies of membrane proteins will provide information about the structure of membrane proteins and the relationship between their structural dynamics and function (Tsukazaki et al., 2011; Tanaka et al., 2015; Bolla et al., 2019). These studies will also help in the development of drugs for various membrane protein-related diseases. Through the understanding of membrane proteins, researchers gradually realized the need for some biological membrane mimetics for the physiologically and pathologically relevant researches (Dufourc, 2021). In the past few decades, although detergents and liposomes have been the traditional methods for reconstructing membrane proteins, the emergence of phospholipid nanodiscs (LNDs) has provided researchers with better tools for studying membrane proteins (Krishnarjuna and Ramamoorthy, 2022). Figure 1B shows the general process of antibody screening using nanodiscs as antigens for membrane proteins.

### 3.1 Drug discovery of therapeutic membrane protein

In a study of the role of autoantibodies against skeletal muscle acetylcholine receptors (AChR) in the pathogenesis of the autoimmune disease myasthenia gravis (MG), Purified AChR protein was successfully incorporated into a membrane scaffold protein nanodisc (MSPND)/phospholipid structure (Sheng et al., 2010). The intravenously administered MSPND-AChR complex was shown to be effective in downregulating anti-AChR antibodies *in vivo* and subsequently improving the pathological outcomes in MG experimental mouse models, which provides a direction for the treatment of this disease.

The emergence of SARS-CoV-2 variants that evade vaccines has prompted the need for vaccines that can trigger widely neutralizing antibodies (bnAb). Mahmoud L Nasr et al. constructed several variants of recombinant SARS-CoV-2 spike glycoprotein decorated cNDs to induce bnAb through vaccination (Mabrouk et al., 2023). Cobalt porphyrin phospholipid (CoPoP) was incorporated into the nanodisk to allow the spike trimer to anchor and functionally orient on the surface of the nanodisk through its His tag. Monophosphoryl lipid A (MPLA) and QS-21 were added as immunostimulatory adjuvants to enhance vaccine response. After optimizing the assembly of nanodisks, spike proteins are effectively displayed on the surface of the nanodisks and maintain their conformational ability to bind to human angiotensin-converting enzyme 2 (hACE2). These NDs vaccines can trigger a wide range of neutralizing antibodies that can neutralize mismatched viruses the following year, thereby reducing immune evasion against newly emerging variants and enhancing healthcare preparedness. And in another patent, researchers assembled hACE2 into SMA nanodisks with antiviral properties (KWEON et al., 2024).

In an ongoing study of neurodegenerative diseases.α-Synuclein (a-Syn), the primary cause of Parkinson’s disease, is associated with lipid changes, researchers used nanodiscs containing lipids with various charges and acyl chain saturation to show that cholesterol has a general inhibitory activity on lipid vesicles. The researchers also created and analyzed the effects of cholesterol-containing nanodiscs in recent studies. In addition, the incorporation of cholesterol and PE into SMA nanodiscs proved to be a different model system. Nanodiscs eliminate the need for unstable and insoluble vesicles in a-Syn fibrillization experiments, allowing for longer experimental durations. In addition, the folding-reducing properties of cholesterol-containing lipid nanodiscs, which have been found to act as a significant promoter of a-Syn fibrillization, are expected to play a key role in the treatment of Parkinson’s disease.

A market research reports point out its great potential in commercial applications. For example, adsorption of nanodiscs on nanosized cell surfaces, binding of chemically synthesized lipids (i.e., biotinylation) to high affinity sites on the streptavidin surface, or interaction through genetically modified or chemically altered sites on histidine tags or msp bands, can sometimes retain membrane proteins in their natural bilayer environment, but do not affect activity when close to the electrode or sensor surface.

### 3.2 Drug discovery of antibodies against membrane protein

Endothelin receptor A (ETA), a class A GPCR, is involved in the progression and metastasis of colorectal cancer, breast cancer, lung cancer, ovarian cancer, and prostate cancer. Human ETA was overexpressed and purified in *E. coli*, and reconstituted with lipids and MSP to prepare ETA-nanodiscs as functional antigens. A single-chain variable fragment (scFv) phage library was constructed and screened, and an antibody (AG8) with high specificity and affinity for ETA was successfully isolated (Ju et al., 2021). AG8 treatment reduced the ETA-induced phosphorylation of protein kinase B and extracellular regulated kinase. In addition, the study showed that AG8 effectively inhibited tumor growth in a colorectal cancer xenograft mouse model.

Parathyroid hormone receptor 1 (PTH1R) is also a protein belonging to G protein coupled receptors (GPCRs). The binding of its ligand to PTH1R involves binding to the large extracellular domain (ECD) and ortho pocket, inducing conformational changes in the transmembrane domain and receptor activation. PTH1R regulates bone metabolism mainly through G_s_ and G_q/11_G proteins. Kaushik Sarkar et al. used SMA nanodisks to display PTH1R ECD as an antigen on bacteriophages and identified ECD scFvhFc (Sarkar et al., 2019). ECD scFvhFc may be a valuable tool for studying PTH1R signal bias.

Developing antibody agonists targeting the human apelin receptor (APJ) is a promising therapeutic approach for treating chronic heart failure. Yanbin Ma et al. reconstructed thermally and conformally stable APJ proteins in nanodisks (Ma et al., 2020). APJ nanodisks are used as immunogens to generate immune pools in camels. Using APJ protein liposomes as antigens, APJ specific sdAbs were isolated from the camel immune sdAb library through phage display. This strategy generated 186 unique sdAbs, among which one of the most effective antagonistic sdAbs is JN241. Through the analysis of complex structures and other works, it has been proven that JN241 is an effective competitive antagonist of human APJ, providing a solution for the treatment of chronic heart failure.

In another study on antibody drug development for matrix protein 2 (M2) of influenza A virus, M2 (1–46) was incorporated into nanodiscs (M2-NDs) to form a membrane-embedded tetrameric conformation, similar to its natural physiological state in the influenza virus envelope (Yu et al., 2023). *Chiloscyllium plagiosum* (Whitespotted bamboo shark) immunization was then performed. Functional vNARs were selected from the shark immune library by phage display technology. One of the isolated vNARs, AM2H10, can specifically bind to tetrameric M2. In addition, AM2H10 was validated for its effectiveness against influenza virus by blocking ion influx through adamantane-sensitive and -resistant M2 channels.

## 4 Lipid nanodiscs for drug delivery

Due to its unique 1) high biocompatibility: lipid nanodiscs mimic the structure of natural cell membranes, with good biocompatibility and low toxicity 2) drug loading capacity: lipid nanodiscs can efficiently loads and protect hydrophobic and hydrophilic drug molecules (Figure 1Ca) 3) Stability and controllability: By selecting different lipids and stabilizers, the size, shape and surface properties of the nanodiscs can be controlled to improve the stability and delivery efficiency of the drug (Figure 1Cb) 4) Target tropism and functionalization: Lipid nanodiscs can achieve targeted delivery to specific cells or tissues through surface modification of targeting molecules (such as antibodies or peptides) (Figure 1Cc). Lipid nanodiscs have shown great application potential in the field of drug delivery and have become research hotspots in the field of drug delivery. Its main applications include small molecular drug delivery, peptide delivery, gene drug delivery, and vaccine delivery.

### 4.1 Small molecular drug delivery

Insufficient drug delivery to the tumors is a major challenge in anticancer drug treatment. Lipid nanodiscs, due to its non-spherical nature, have demonstrated prolonged plasma half-life and higher cellular internalization compared to the traditional liposome carrier (Bariwal et al., 2022). Lipid nanodisc could be used to deliver anticancer drug such as immune-oncology drug STING agoinst. Activation of the stimulatory factor of innate immunity (STING) pathway of interferon genes can enhance anti-tumor immunity, but systemic delivery of STING agonists to tumors is extremely challenging. Dane et al., developed a method to couple cyclic dinucleotides (CDN) STING agonists to polyethylene glycol (PEG)-lipids via cleavage linkers and incorporate them into lipid nanodiscs (LND) (Dane et al., 2022). Intravenously administered LNDs containing CDN-PEG-lipids penetrate tumors more efficiently, exposing the majority of tumor cells to the effects of STING agonists. A single dose led to tumor regression with immune memory associated with anti-tumor re-challenge. The cancer cell uptake is associated with powerful T cell activation by promoting the co-localization of CDN and tumor antigens in dendritic cells. LNDs thus serve as a vehicle that holds promise for robust delivery of compounds throughout solid tumors, which could be used to enhance immunotherapy. Another study showed that doxorubicin (DOX)-loaded LNDs enhanced immune checkpoint blockade in a mouse tumor model and triggered immunogenic cell death (ICD) of cancer cells and exerted antitumor efficacy without significant off-target side effects (Kuai et al., 2018). Priming tumors using DOX-carrying nanodiscs elicited robust antitumor CD8^+^ T cell responses while expanding their epitope recognition of tumor-associated antigens, neoantigens, and intact tumor cells. Furthermore, combination of DOX-carrying nanodiscs and anti-PD1 therapy induced complete regression of established CT26 and MC38 colon cancer tumors in more than 80% of animals and protected survivors from tumor recurrence. The results provide a new framework for using nanodisc-based chemotherapy to prime anti-tumor immunity and sensitize tumors to immune checkpoint blockade.

Besides oncology application, lipid nanodiscs could also be used to deliver small molecules for potential treatment of metabolic diseases, retinal diseases, or neurological diseases, etc. He et al., have developed synthetic HDL nanodiscs with a renal-targeting KT peptide to specifically deliver liver X receptor (LXR) agonist to the renal mesangial cells in mouse model of diabetic nephropathy (DN) for the suppression of cell proliferation, lipid accumulation and inflammation, demonstrating promising efficacy and minimizing the hepatic toxicity (He et al., 2022). Retinal protecting Lutein is poorly soluble and could not be absorbed very efficiently by oral administration. Nanodiscs have been used to incorporate Lutein to enhance its cellular uptake for the protection of photoreceptor cells from UV-induced damage (Moschetti et al., 2022). The polyphenolic α-mangiferin (α-M) has been successfully loaded onto APOE-reconstituted nanodiscs, which demonstrated good brain blood barrier (BBB) penetration and accumulation around Aβ aggregates in SAMP8 AD model mice (Song et al., 2016). α-M-NDs bind strongly to Aβ, effectively reduce amyloid plaque deposition and improve the neurological deficits.

### 4.2 Peptide drug delivery

In addition to small molecule drugs, peptide drugs could also be efficient incorporated and delivered by lipid nanodiscs. Song et al. has used GM1 modified nanodiscs to load neuroprotective NAP peptide for intranasal administration in AD model mice and observed efficient distribution of NAP peptide in the mice brain, reduced Aβ aggregate deposition and amelioration of neurological deficits, such as memory loss (Song et al., 2016). For the half-life extension of Substance P (SP) peptide due to the cleavage by neutral endopeptidase (NEP), SP was incorporated in nanodiscs to improve its *in vitro* and *in vivo* stability with greater than 3 order of magnitude prolongation of SP half-life (Huang et al., 2015). In this way, the retention of the SP peptide in bone marrow is improved, with enhanced angiogenesis and restoration of blood perfusion in a diabetic limb ischemia model.

### 4.3 Gene drug delivery

Lipid nanodiscs can effectively deliver gene drugs (such as DNA, RNA) and improve their expression efficiency and stability within cells. Lipid nanodiscs can protect genetic drugs from degrading enzymes in the body, improving their stability and delivery efficiency in the body. siRNA is an important gene silencing tool, but it has poor stability in the body and is easily degraded. By embedding small interfering RNA (siRNA) into lipid nanodiscs, researchers were able to significantly improve its intracellular delivery efficiency and gene silencing effect. RGD motif has long been exploited as a versatile tool for targeted drug delivery (Liang et al., 2023). Chen et al., prepared lipid nanodiscs (LNDs) with cyclic RGD peptide (cRGD) on either edges or planes, respectively, to design two anisotropic targeting nanocarriers (E-cRGD-NDs and P-cRGD-NDs) for siRNA delivery (Chen et al., 2020). The results showed that E-cRGD-NDs showed obvious advantages in terms of siRNA loading, cellular uptake, silencing efficiency, protein expression and *in vivo* effects. This has been attributed to that the edge modification of cRGD effectively separates the targeting domain and siRNA loading domain in LNDs, avoiding mutual interference between different components and maximizing their respective functions.

### 4.4 Vaccine delivery

LNDs demonstrate superiority in vaccine delivery. They can display antigens, enhance the body’s immune response, and increase the effectiveness of vaccines. LNDs can be used as vaccine carriers through surface modification of antigenic proteins or peptides to improve the immunogenicity and protective effect of vaccines. Guo et al., has generated LNDs from cancer cell membrane with enhancement of lipid-based adjuvant as cancer vaccine. LNDs containing cancer antigen and adjuvant are taken up by APC cells to simulate the immune system for the recognition and killing of cancer cells, demonstrating good anti-tumor efficacy in CRC model and melanoma model. (Guo et al., 2023).

## 5 Outlook

Although lipid nanodiscs show great potential in membrane protein drug discovery & development as well as drug delivery, they still face some challenges: 1) How to achieve efficient and low-cost large-scale preparation is a key issue for the application of lipid nanodiscs. 2) The metabolism and degradation mechanism of lipid nanodiscs in the body needs further study to ensure their stability, safety and effectiveness in the body. The interaction mechanism between lipid nanodiscs and biomolecules still needs to be further studied to optimize their application.

Different nanodiscs also have some shortcomings. MSP nanodiscs have characteristics that real biological membranes do not have. First, they cannot simulate membrane curvature, and second, they cannot simulate the asymmetry of lipids in cell membranes - both of which have some influence on regulating the binding of peripheral membrane proteins. Saposin nanodiscs are less used, mainly because they are in an awkward situation. Compared with MSP nanodiscs, they are not as uniform and stable as MSP nanodiscs. Compared with peptidisc and copolymer nanodiscs, their assembly process is more complicated.Compared with other types of nanodiscs systems, the cost of custom synthetic scaffold peptides is relatively high, and they have lower stability due to their non-covalent assembly, which is the main disadvantage of peptidisc. For some nanodiscs constructed by SMA, the first is biosafety, and SMA has strong absorbance in the ultraviolet region, which affects the reconstructed membrane proteins. Secondly, SMA is unstable at low pH and in the presence of divalent metal ions, and is easily precipitated in the presence of divalent cations (including Mg2+ and Ca2+) [201]. In addition, the solubilization efficiency of SMA is generally lower than that of commonly used detergents [201]. Finally, the negative charge on SMAs can interfere with the binding of tags to affinity resins, particularly during metal affinity chromatography [198]. Although several new polymer derivatives have been recently developed that reduce these disadvantages, the development of polymer nanodiscs remains an area of ongoing development.

The future development directions of lipid nanodiscs include 1) Multifunctional nanodiscs: By surface modification of multiple functional molecules (such as targeting molecules, fluorescent probes, etc.), multifunctional lipid nanodiscs can be developed to achieve simultaneous diagnosis and treatment 2) Smart nanodiscs: Develop smart lipid nanodiscs that can respond to specific physiological conditions (such as pH, temperature) to achieve controlled release of drugs 3) Personalized drug delivery: Lipid nanodiscs based Drug delivery systems can be personalized according to the patient’s specific conditions to improve treatment effectiveness.
